# Renoprotective Effects of Metformin are Independent of Organic Cation Transporters 1 & 2 and AMP-activated Protein Kinase in the Kidney

**DOI:** 10.1038/srep35952

**Published:** 2016-10-26

**Authors:** Michael Christensen, Jonas B. Jensen, Steen Jakobsen, Niels Jessen, Jørgen Frøkiær, Bruce E. Kemp, Allison L. Marciszyn, Hui Li, Núria M. Pastor-Soler, Kenneth R. Hallows, Rikke Nørregaard

**Affiliations:** 1 Department of Clinical Medicine, Aarhus University, Denmark; 2 Department of Nuclear Medicine and PET Centre, Aarhus University Hospital, Denmark; 3 St. Vincent’s Institute of Medical Research, University of Melbourne, Mary MacKillop Institute for Health Research Australian Catholic University, Victoria Parade, Fitzroy VIC 3065, Australia; 4 Renal-Electrolyte Division, Department of Medicine, University of Pittsburgh School of Medicine, Pittsburgh, PA, USA; 5 Division of Nephrology and Hypertension, Department of Medicine and USC/UKRO Kidney Research Center, University of Southern California Keck School of Medicine, Los Angeles, CA, USA

## Abstract

The type-2 diabetes drug metformin has proven to have protective effects in several renal disease models. Here, we investigated the protective effects in a 3-day unilateral ureteral obstruction (3dUUO) mouse model. Compared with controls, ureteral obstructed animals displayed increased tubular damage and inflammation. Metformin treatment attenuated inflammation, increased the anti-oxidative response and decreased tubular damage. Hepatic metformin uptake depends on the expression of organic cation transporters (OCTs). To test whether the effects of metformin in the kidney are dependent on these transporters, we tested metformin treatment in OCT1/2^−/−^ mice. Even though exposure of metformin in the kidney was severely decreased in OCT1/2^−/−^ mice when evaluated with [^11^C]-Metformin and PET/MRI, we found that the protective effects of metformin were OCT1/2 independent when tested in this model. AMP-activated protein kinase (AMPK) has been suggested as a key mediator of the effects of metformin. When using an AMPK-β1 KO mouse model, the protective effects of metformin still occurred in the 3dUUO model. In conclusion, these results show that metformin has a beneficial effect in early stages of renal disease induced by 3dUUO. Furthermore, these effects appear to be independent of the expression of OCT1/2 and AMPK-β1, the most abundant AMPK-β isoform in the kidney.

The main function of metformin, a biguanide compound widely used for treatment of type 2 diabetes mellitus, is to lower the level of blood glucose^1^,^2^ by inhibiting hepatic gluconeogenesis^3^,^4^,^5^ and increase cellular glucose uptake^6^,^7^. Furthermore, metformin may decrease cardiovascular complications for these patients^8^,^9^. Metformin has also been tested in various other disease models where it has been shown to have anti-oncogenic^10^,^11^, cardioprotective^12^,^13^, and anti-inflammatory effects^14^,^15^.

Metformin has been shown to attenuate diabetic nephropathy (DN) when tested in a streptozotocin-induced DN model, possibly by upregulating the anti-oxidative response^16^. Recently, it has also been demonstrated that metformin attenuates progression of fibrosis in dogs subjected to 14 days unilateral ureteral obstruction (UUO)^17^, as well as in mouse models of 7 and 14 days UUO^18^,^19^.

The beneficial effects of metformin in type 2 diabetes are partly due to its effects in the liver, where organic cation transporters 1 (OCT1) is responsible for the metformin uptake into the hepatocytes^20^. Genetic ablation of OCT1 in mice impairs the glucose lowering effects of metformin^21^. Organic cation transporters 1 and 2 (OCT1/2) belong to the SLC22 transporter family that eliminate waste and toxic compounds via the kidney^22^. Both transporters are expressed at the basolateral membrane of the proximal tubules in the kidney of rodents^23^. Humans express only OCT2 in the kidney, and therefore the double OCT1/2^−/−^ mice might be the better model for evaluating the significance of this transport system in the kidney^24^,^25^. Recently OCT3 has also been suggested to be implicated in the pharmacologic response to metformin^26^,^27^. OCT3 is also expressed in the kidney, although to a much lower extent compared to OCT1 and OCT2^27^.

Metformin is a known activator of AMP-activated protein kinase (AMPK)^28^, and AMPK has been suggested as a key molecule in the renoprotective effects of metformin (e.g., in an ischemia reperfusion model)^29^. In addition, metformin-dependent activation of AMPK inhibits the production of TGF-β induced fibrosis in primary renal fibroblasts^30^.

Based on these considerations, we hypothesized that metformin would have anti-inflammatory as well as protective effects against tubular injury in response to 3dUUO. We further tested the dependency of these effects on the expression of OCT1/2 and AMPK.

## Results

### Effect of metformin on renal function in response to 3dUUO

To examine the effects of metformin in an obstructive nephropathy model, mice received metformin in their drinking water (500 mg/kg/day) for 7 days prior to 3dUUO as well as during the obstruction. Metformin-treated mice displayed elevated plasma metformin levels, suggesting that the oral route of administration was successful. Plasma metformin levels were higher in the UUO compared to SHAM, indicating impairment of renal metformin clearance. Plasma creatinine and urea plasma levels were increased in UUO compared to SHAM mice. Metformin treatment did not affect these parameters (Supplementary data, Table 1).

### Metformin prevents Inflammation in response to 3dUUO

**Figure 1:**
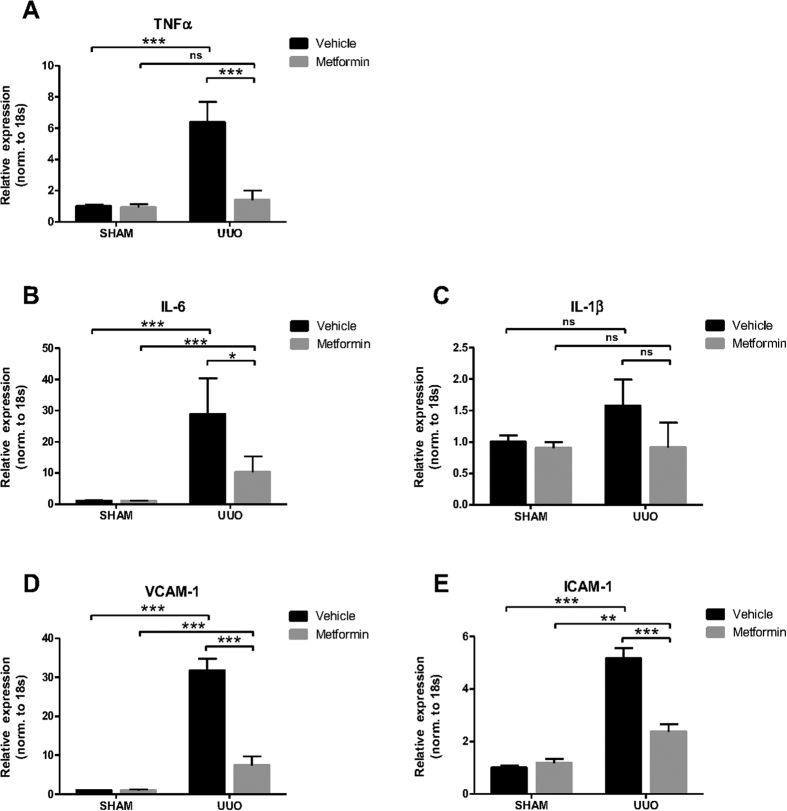
Metformin prevents inflammation in response to 3dUUO. (**A–C**) Regulation of the inflammatory markers TNFα, IL-6 and IL-1β in response to 3dUUO and metformin treatment normalized to ribosomal 18S RNA. (**D,E**) The regulation of the VCAM-1 and ICAM-1 following UUO and metformin treatment normalized to ribosomal 18S RNA. Each bar represents the mean ± SEM. P < 0.05 was considered statistically significant indicated by *P < 0.01 by **P < 0.001 by ***, and not significant by ns.

**Figure 2:**
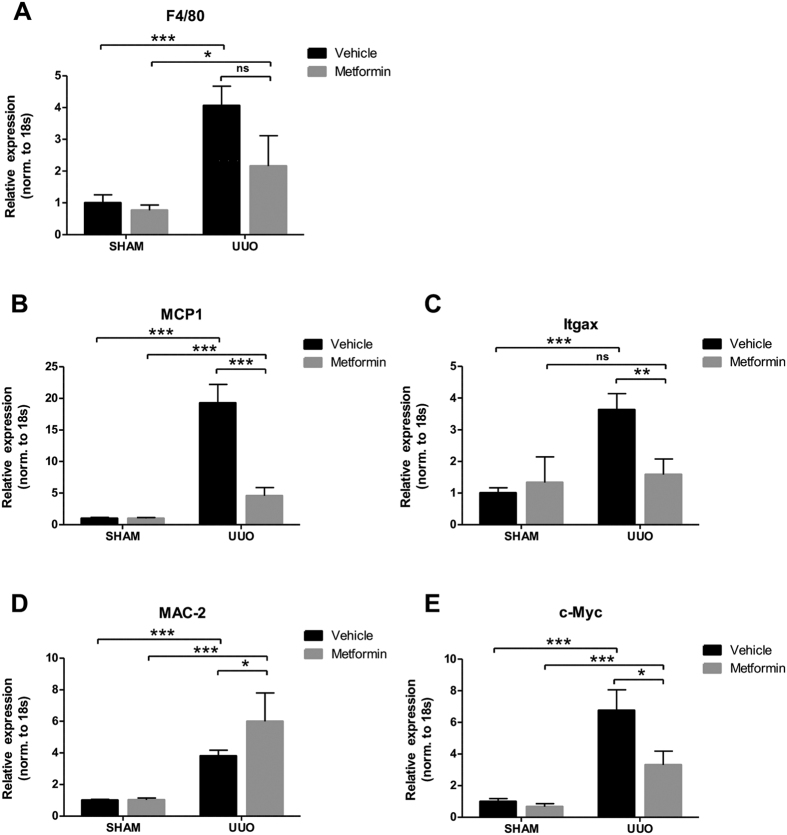
Metformin plays a role for the regulation of macrophage subpopulations under UUO. (**A**) Regulation of the general macrophage marker F4/80 normalized to ribosomal 18S RNA. (**B,C**) Pro-inflammatory marker MCP-1 and Itgax normalized to 18S RNA. (**D**,**E**) Regulation of the anti-inflammatory marker MAC-2 and c-Myc normalized to 18S. Each bar represents the mean ± SEM. P < 0.05 was considered statistically significant indicated by *P < 0.01 by **P < 0.001 by ***, and not significant by ns.

To investigate the effect of metformin on renal inflammation in response to 3dUUO, we measured the mRNA expression of tumor necrosis factor alpha (TNFα), interleukin 6 (IL-6) and interleukin 1beta (IL-1β). Mice subjected to UUO showed increased levels of these inflammation markers compared to SHAM mice. Metformin treatment prevented the upregulation of TNFα, IL-6 and IL-1β in UUO mice, although IL-1β did not reach statistical significance (Fig. 1A–C). Intercellular adhesion molecule 1 (ICAM-1) and vascular cell adhesion protein 1 (VCAM-1) play a role for the recruitment of leukocytes to the sites of injury^31^,^32^. The expression of these mRNAs were increased in response to UUO, which was attenuated by metformin treatment (Fig. 1D,E). The general marker for macrophages, F4/80 showed higher mRNA expression in UUO compared to SHAM mice, but was not significantly downregulated in metformin treated UUO mice, although there was a trend (Fig. 2A). To further analyze the heterogeneity of the macrophages, we analyzed the mRNA expression in the kidney tissue of the pro-inflammatory M1 specific macrophage marker Monocyte Chemoattractant Protein-1 (MCP-1) and Integrin alpha X (Itgax) (Fig. 2B,C) and the anti-inflammatory M2 specific marker *macrophage* galactose-specific lectin-2 (Mac-2) and c-Myc (Fig. 2D,E). Both M1 and M2 macrophages were significantly increased in the UUO compared to SHAM mice. Both M1 macrophages MCP-1 and Itgax as well as the M2 macrophage c-Myc were downregulated, whereas Mac-2 was upregulated in the metformin treated animals. The metformin-dependent downregulation of the above mentioned inflammatory markers suggest that metformin has anti-inflammatory effects in response to 3dUUO and potentially play a role for the regulation of the subpopulations of M1 and M2 macrophages.

### Metformin prevents tubular injury and increases the anti-oxidant response in 3dUUO

**Figure 3:**
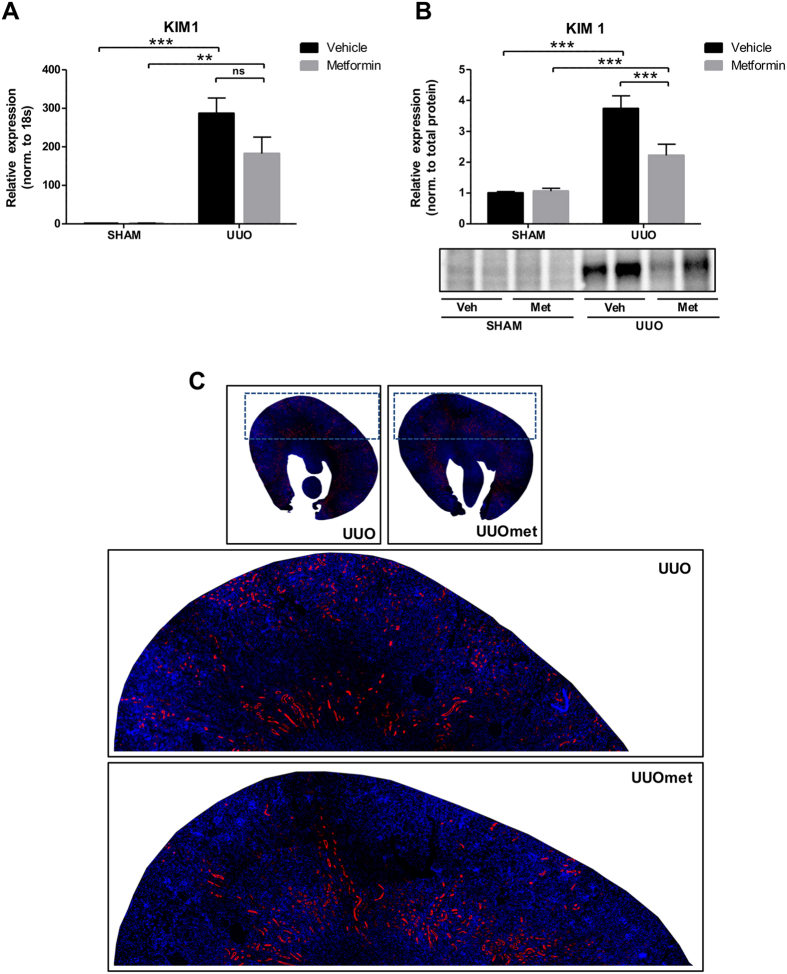
Metformin attenuates the expression of KIM-1 following UUO. (**A**) The regulation of KIM-1 mRNA normalized to 18S RNA. (**B**) Regulation of KIM-1 protein level normalized to total protein. (**C**) Representative whole kidney sections stained for KIM-1 (red) and nuclei (blue). Each bar represents the mean ± SEM. P < 0.05 was considered statistically significant indicated by *P < 0.01 by **P < 0.001 by ***, and not significant by ns.

**Figure 4:**
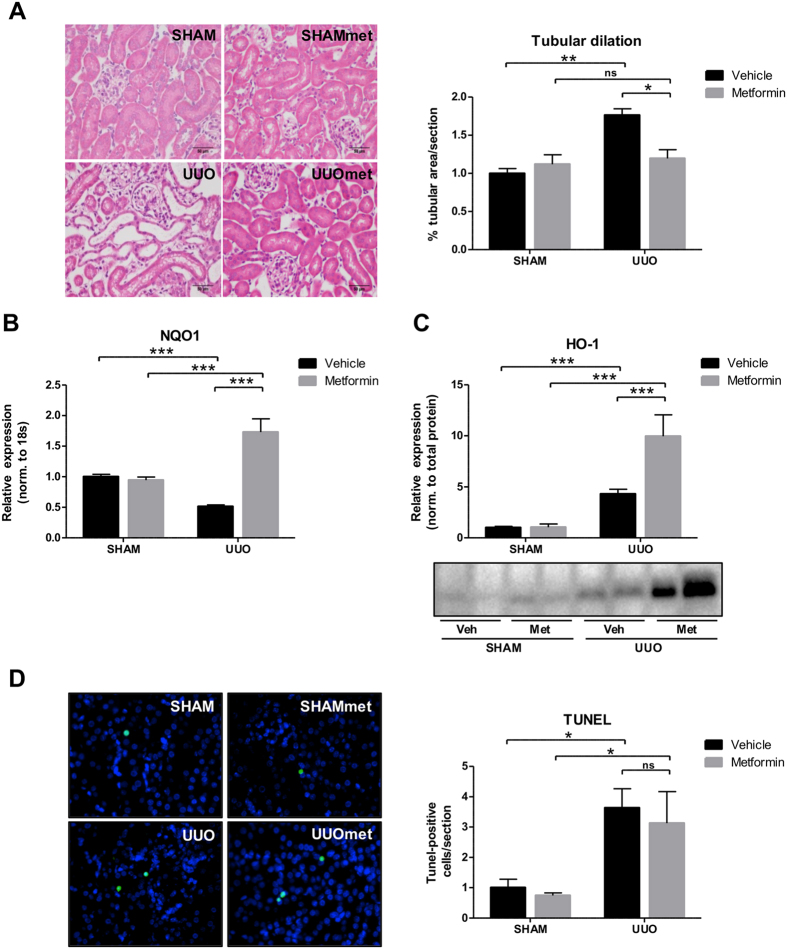
Metformin attenuates tubular dilation, increases the antioxidant response, but does not affect the level of apoptosis. (**A**) Representative H&E staining and quantitative analysis of the level of tubular dilation in the cortex. (**B**) Regulation of NQO1 at mRNA level in response to UUO normalized to 18S RNA. (**C**) Protein expression of HO-1 normalized to total protein level. (**D**) TUNEL assay with TUNEL-positive cells as green, and nuclei as blue. *Right*, a quantification of the number of TUNEL-positive cells/section. Each bar represents the mean ± SEM. P < 0.05 was considered statistically significant indicated by *P < 0.01 by **P < 0.001 by ***, and not significant by ns.

Kidney injury molecule-1 (KIM-1) is a specific biomarker for kidney proximal tubule injury^33^. KIM-1 mRNA and protein expression were increased in mice subjected to UUO compared to SHAM mice. Metformin treatment attenuated the increased KIM-1 at both the mRNA and protein levels, although it did not reach statistical significance at the mRNA level (Fig. 3A,B). To evaluate the cortical localization of KIM-1, we performed fluorescence microscopy, which showed that KIM-1 was mostly downregulated in the outer part of the cortex in metformin-treated UUO mice (Fig. 3C). To further investigate the level of tubular injury, we performed hematoxylin and eosin (H&E) staining of whole kidney sections. We quantified cortical tubular dilation and found that the tubules were significantly dilated in UUO compared to SHAM mice, but this dilation was significantly attenuated in metformin-treated UUO mice (Fig. 4A). Antioxidants play an essential role in reducing levels of reactive oxidant species (ROS) produced during UUO, with an increase in antioxidants potentially limiting the damage created by ROS. Metformin increased both the cytoprotective antioxidant NAD(P)H quinone oxidoreductase 1 (NQO1) mRNA expression and HO-1 protein levels in mice subjected to UUO, indicating that metformin upregulates the antioxidative response to 3dUUO (Fig. 4B,C). The level of apoptosis was evaluated using a TUNEL assay. In response to UUO, there was a significant increase in TUNEL positive nuclei, consistent with increased apoptosis. Metformin treatment had no effect on the degree of apoptosis in UUO mice (Fig. 4D). These data indicate that metformin decreases tubular injury and increases the antioxidant response to 3dUUO but does not affect the level of apoptosis.

### The renoprotective effects of metformin are OCT1/2 independent

**Figure 5:**
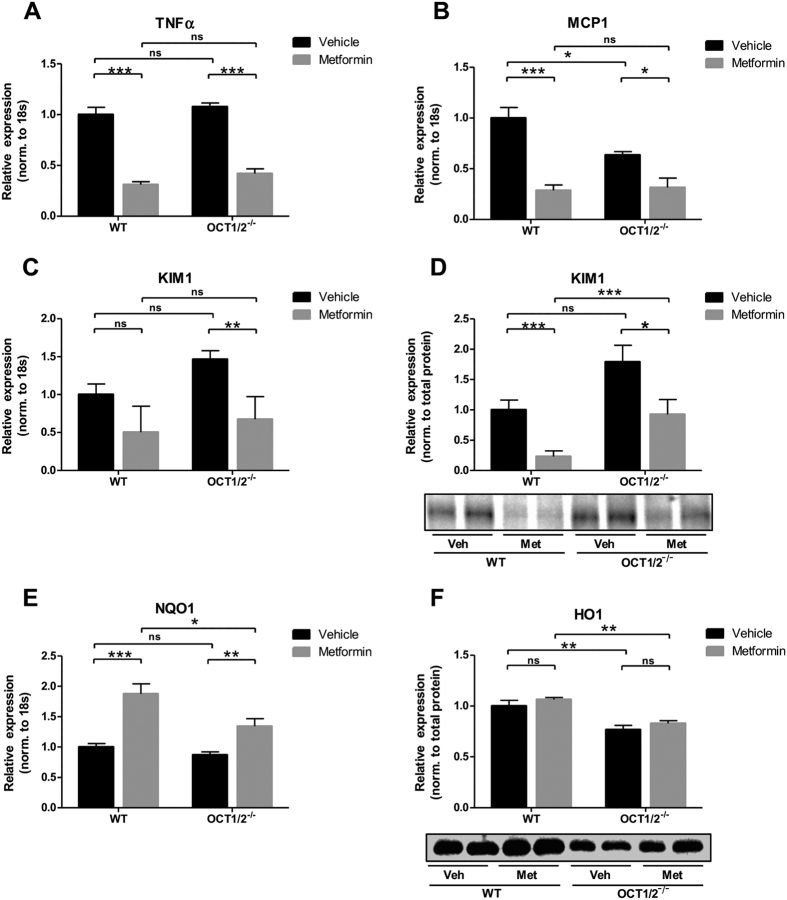
The renoprotective effects of metformin are independent of the expression of OCT1/2. (**A**,**B**) mRNA regulation of TNFα and MCP-1 normalized to 18S. (**C**) regulation of mRNA of KIM-1 normalized to 18S. (**D**) Protein expression of KIM-1 normalized to total protein. (**E**) Regulation of NQO1 at mRNA level normalized to 18S. Regulation of HO-1 protein expression normalized to total protein. Each bar represents the mean ± SEM. P < 0.05 was considered statistically significant indicated by *P < 0.01 by **P < 0.001 by ***, and not significant by ns.

To test the hypothesis that the renoprotective effects of metformin in response to 3dUUO are dependent on OCT1/2 expression, WT and OCT1/2^−/−^ mice were subjected to UUO, and half of the mice in each group were treated with metformin. Plasma metformin concentration was increased in OCT1/2^−/−^ metformin-treated mice compared to WT. Plasma creatinine was also elevated in OCT1/2^−/−^ mice compared to WT, and further elevated in metformin-treated OCT1/2^−/−^ mice, whereas plasma urea levels were lower in both groups of OCT1/2^−/−^ mice compared to WT mice (Supplementary data, Table 2). TNFα and MCP-1 mRNA expression were reduced after metformin administration to both the WT and OCT1/2^−/−^ UUO mice compared to vehicle-treated mice (Fig. 5A,B). Interestingly, MCP-1 was expressed at a lower level in OCT1/2^−/−^ vehicle-treated mice compared to WT vehicle-treated mice. KIM-1 mRNA and protein expression were decreased in both WT and OCT1/2^−/−^ mice treated with metformin (Fig. 5C,D). NQO1 mRNA expression was increased in the metformin treated groups, which was evident both in WT and OCT1/2^−/−^ mice (Fig. 5E). HO-1 protein levels were not modulated by metformin treatment in either WT or OCT1/2^−/−^ mice (Fig. 5F). These data suggest that the renoprotective effects of metformin are independent of OCT1/2 expression.

**Figure 6:**
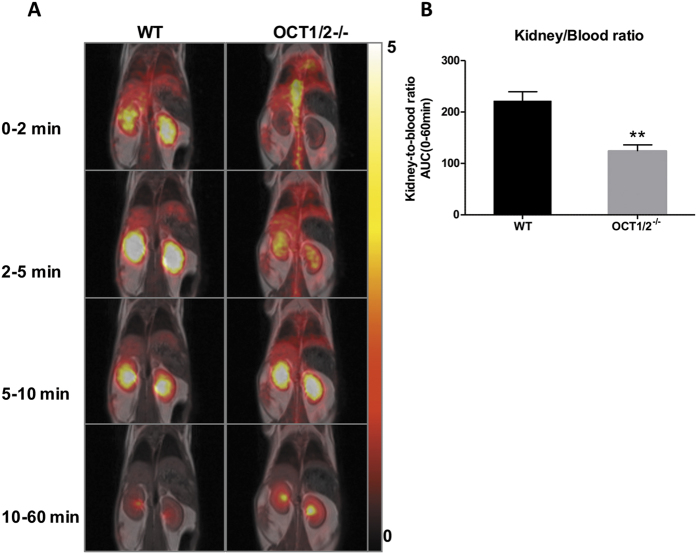
Distribution of [^11^C]-metformin in the kidneys. (**A**) Coronal PET and corresponding MR-image in a wild type (WT) and an OCT1/2^−/−^ mouse during different time intervals. Scale bar to the right represents Stardard Uptake Value (SUV) = concentration [kBq/ml] x (body weight [g]/injected dose [kBq]. B: Kidney-to-blood ratio AUC of [^11^C]-metformin from 0-60 min. Black bar = WT (n = 6); gray bar = OCT1/2^−/−^ (n = 4). Each bar represents the mean + SEM. P < 0.01 is indicated by **.

**Figure 7:**
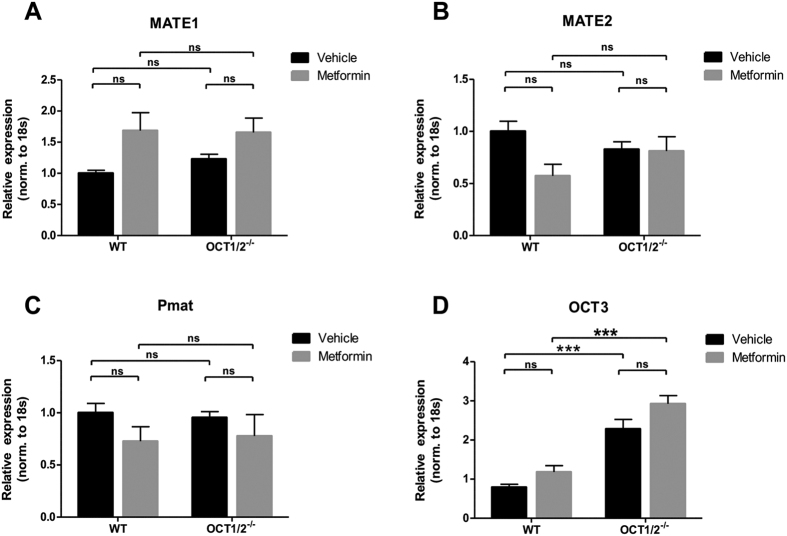
Regulation of metformin transporters in kidney cortex. (**A**–**D**) mRNA expression of Mate1, Mate2, Pmat and OCT3 normalized to 18S. Each bar represents the mean ± SEM. P < 0.05 was considered statistically significant indicated by *P < 0.01 by **P < 0.001 by ***, and not significant by ns.

To test whether there were any differences between the accumulation of metformin in the kidney between the OCT1/2^−/−^ and WT animals, [^11^C]-metformin was injected intravenously into mice, and the distribution of metformin in the kidney was evaluated as the area under the kidney-to-blood ratio curve during 60 min (Fig. 6A,B). The results show that there was significantly less metformin accumulation in the kidneys of OCT1/2^−/−^ mice compared to WT mice, suggesting less cellular uptake of metformin in the OCT1/2^−/−^ mice. To test whether other proteins might compensate for the ablation of OCT1/2, the expression of the metformin transporter proteins multidrug and toxin extrusion 1 and 2 (Mate-1 and Mate-2), OCT3 and plasma membrane monoamine transporter (Pmat) were analyzed in both WT and OCT1/2^−/−^ mice (Fig. 7). Neither, Mate1, Mate2 nor Pmat were significantly altered in the WT and OCT1/2^−/−^ mice after metformin treatment (Fig. 7A–C). In contrast, OCT3 mRNA expression was increased in OCT1/2^−/−^ compared to WT mice (Fig. 7D).

### The renoprotective effects of metformin are not affected by the KO of AMPK-β1

**Figure 8:**
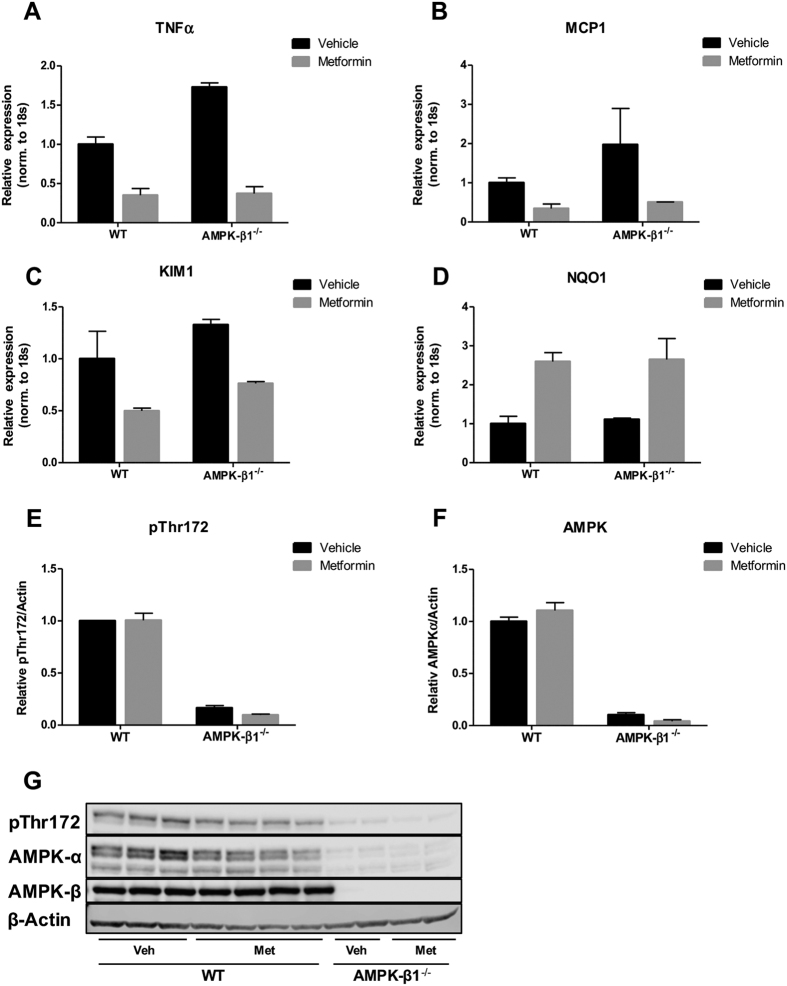
The renoprotective effects of metformin are independent of AMPK-β1. (**A–D**) Regulation of the inflammatory markers TNFα and MCP1, the injury marker KIM-1 and antioxidant NQO1 normalized to ribosomal 18S RNA. Each bar represents the mean ± SEM. (**E**,**F**) Protein regulation of AMPK-α and AMPK-α phospho-Thr172 normalized to β-actin. Each bar represents the mean ± SEM. (**G**) Western blots of AMPK-α, AMPK-α phospho-Thr172 and AMPK-β1. N-value 2-4 animals.

As AMPK has been suggested to be implicated in the renoprotective effects of metformin^34^, we next tested the potential role of AMPK in the 3dUUO model of renal damage using AMPK-β1^−/−^ mice. The AMPK-β1^−/−^ model was used for these experiments because KO mouse was found to practically ablate the expression of AMPK active enzyme in mouse kidney^35^. The inflammatory markers TNFα and MCP1, as well as KIM-1, showed downregulation in the metformin-treated WT as well as AMPK-β1^−/−^ animals (Fig. 8A–C). In addition, the antioxidant NQO1 was increased in response to metformin treatment (Fig. 8D). Western blotting revealed that metformin did not affect the level of activated AMPK (AMPK-α-pThr172) or AMPK-α, but both were dramatically suppressed in AMPK-β1^−/−^ mice as compared to WT mice (Fig. 8E–G). These data suggest that the renoprotective effects of metformin are not dependent specifically on functional AMPK expression in kidney tissue.

## Discussion

The main results of the present study demonstrate that metformin treatment attenuates development of renal inflammation and tubular damage in the obstructed kidney in mice subjected to 3dUUO. These results are consistent with a previous report where metformin treatment of UUO for 7 and 14 days attenuated the level of inflammatory markers TNFα and VCAM1^19^. Furthermore, we observed differential changes in expression of the anti-inflammatory macrophage marker Mac-2 and the pro-inflammatory markers MCP-1 and Itgax, which increased and decreased respectively, with metformin treatment, indicating that metformin might play a role for the regulation of the subpopulations of macrophages. It has previously been described in monocytes that a shift toward an anti-inflammatory macrophage phenotype occurred when this primary cell culture was treated with metformin^36^.

KIM-1 has been demonstrated to be a proximal tubular injury marker^33^. In the present study we demonstrate a significant downregulation of KIM-1 in the metformin-treated UUO mice. We also used tubular dilation as a measure of tubular damage and found decreased dilation in the metformin-treated UUO mice. This finding support the results of previous studies demonstrating that metformin can attenuate tubular damage, both in Zucker diabetic fatty (ZDF) rats^37^ and in a gentamicin-induced nephropathy model^38^. A recently published study evaluated the level of tubular dilation in UUO mice treated with metformin in a 7dUUO model, but they were not able to detect any significant attenuation of tubular dilation^19^. This finding may suggest that the metformin has minor effects on tubular dilation at more chronic stages of obstruction. Alternatively, the difference could be explained by differences in the experimental protocol. In the mentioned study mice received metformin 1 day prior to obstruction at a dose of 200 mg/kg/day^19^ as compared with 7 days at 500 mg/kg/day in our study. Surprisingly, metformin did not affect the degree of apoptosis. Metformin has been demonstrated to induce apoptosis in several cancer models^39^,^40^, and there are also examples that metformin treatment can prevent apoptosis^41^. To our knowledge, metformin has not been associated with changes in apoptosis in any other renal disease models. Potentially, we may have been able to detect anti-apoptotic effects in a more chronic obstruction model, but further studies would be needed to test this. Antioxidants can ameliorate UUO-associated injury, as we have previously demonstrated that the antioxidants NADPH oxidase inhibitor diphenyleneiodonium (DPI) and the complex I inhibitor rotenone (ROT) prevented renal damage in response to 3dUUO^42^. Our current data suggest that metformin can upregulate NQO1 and HO-1, which might reduce ROS in response to obstructive nephropathy. The upregulation of HO-1 is consistent with previous results^18^. Interestingly, additional experiments demonstrated that administration of metformin after UUO induction also had some renoprotective effects (Supplementary Figure 1). This finding may potentially have clinical implications. Individuals with UUO rarely suffer from renal insufficiency and consequently there is little risk for developing metformin-associated lactic acidosis^43^.

To investigate the mechanisms responsible for cellular uptake of metformin, which may be involved in the renoprotective effects, we used a mouse model with genetic ablation of both OCT1 and OCT2. OCT1/2^−/−^ mice had higher plasma concentrations of metformin and less metformin accumulation in the kidney evaluated by PET/MRI. However, metformin’s renoprotective effects did not seem to be affected by KO of OCT1/2. Mate-1, Mate-2 and the plasma membrane monoamine transporter (Pmat) are also able to transport metformin, and are localized in the apical membrane of the proximal tubular cells, with Mate-1 being the most abundant^44^,^45^,^46^,^47^. Recent work has suggested that OCT3 has a similar role as Mate-1 in handling metformin in the kidney^27^. In the metformin-treated animals we observed a small but insignificant metformin-dependent upregulation of the apical Mate-1 transporter. Apical transport is the rate-limiting step in excretion of organic cations^48^,^49^. Therefore, treatment with metformin would generate a higher demand for apical transport and thereby Mate-1 expression. The tendency was similar in OCT1/2^−/−^ mice, which could indicate that metformin is still being transported into the proximal tubule cells despite knockdown of OCT1/2. We therefore speculate that metformin is still being transported into the proximal tubule cells, despite the knockdown of OCT1/2. Furthermore, we observed that OCT3 was upregulated in the OCT1/2^−/−^ mice, which most likely works as a compensation for the lack of OCT1/2. A recent study using OCT3^−/−^ mice suggested that OCT3 can have a dual role both at the basolateral and the apical membranes, depending on the load of metformin^27^. It has been demonstrated that OCT1/2^−/−^ results in a 2.5-fold reduction of kidney/plasma concentration of metformin, and that OCT1 and OCT2 collectively account for 60% of the renal uptake^50^. The effects of metformin could be mediated at least partly by OCT3, as the expression of this transporter is increased in the OCT1/2 double-knockout phenotype. Therefore, it is likely that in this constitutive KO model there is a compensation for the lack of OCT1/2. An alternative explanation could be that at least some for the effects are mediated through a systemic effect, where metformin potentially primes the immune system to respond to injury, but examination of this is beyond the scope of this study. Our data suggest that the renoprotective effects might not be dependent on the expression OCT1/2 in this UUO model, and that a compensatory upregulation of OCT3 and potentially other unknown transporters of metformin might be involved.

AMPK has been suggested as a key molecule in the anti-fibrotic effects of metformin^18^,^30^ as well as in preventing tubular cell damage^29^. Metformin has been shown to activate AMPK in C57BL/6 mice subjected to a dose of 300 mg/kg metformin^29^. Other studies have also suggested an involvement of AMPK in the protective effects of metformin^51^,^52^,^19^, where an increase in phosphorylation of AMPK or AMPK activity occurred. Our data suggest that the renoprotective effects are not dependent on the expression of AMPK-β1. Levels of both AMPK-α and AMPK-α-pThr172, markers for AMPK activity were drastically reduced in kidney tissue in the AMPK-β1^−/−^ mice. AMPK-β1^−/−^ mice were earlier tested in an ischemia model, where it was found that the level of AMPK activity was reduced by 96% under basal conditions and by 66% following ischemia as compared to WT^35^. In this study they did not treat with metformin and found no significant effect of AMPK KO on subsequent kidney injury. Our data suggest that the protective effects of metformin are not dependent on the expression of AMPK-β1, which therefore does not support the hypothesis that kidney AMPK is involved in the effects of metformin. We cannot rule out the possibility that AMPK-β2 may be involved in mediating metformin’s action in another tissue. For example, it was recently reported that the inhibition of hepatic gluconeogenesis by metformin was mediated by AMPK in the duodenum via the vagal nerve^5^. One question is whether this KO model is optimal for testing dependency of AMPK, as AMPK activity is dramatically inhibited in the kidney, but may persist in other organs due to the presence and potential upregulation of β2-containing isoforms of AMPK in the AMPK-β1 KO mice^53^,^54^. Indeed, it is possible that the renoprotective effects of metformin may be mediated by AMPK, but are less dependent on AMPK activity in kidney epithelial cells. Moreover, as long as the activity level of AMPK is not completely abolished, we cannot rule out involvement of residual AMPK activity.

In conclusion, the present results suggest that metformin has protective effects when applied in a 3dUUO model regarding both inflammation and tubular injury to the kidney. These effects might be independent of OCT1 and OCT2 transporters when tested in a constitutive OCT1/2^−/−^ model. It is possible that these transporters are sufficient but not essential, as other transporters such as OCT3 or potentially additional transporters are able to compensate for the loss of metformin uptake via OCT1 and OCT2. Moreover, the results obtained using AMPK-β1^−/−^ mice suggest that the renoprotective effects are not dependent on the expression of AMPK in the kidney.

## Methods

### Experimental procedures

Experiments were performed on C57BL/6, FVB, OCT1/2^−/−^ (Taconics, USA) or AMPK-β1^−/−^ mice weighing 20–30 g. The AMPK-β1^−/−^ mice were generated as described^53^. Animals had *ad libitum* access to standard rodent diet and tap water, were kept in cages with 12–12-h light-dark cycle at a temperature within the range of 21 ± 2 °C, and humidity of 55 ± 5%. Animals were anesthetized with sevoflurane (Abbott Scandinavia, Sweden) and placed on a heating pad to maintain normal body temperature. Through an abdominal incision a silk ligature was tied around the left ureter to make a complete obstruction before closure of the animal. Both pre- and post-surgery the animals received analgesic treatment with buprenorphine (Temgesic) (Reckitt Benckiser, England).

#### Protocol 1

Male C57BL/6J mice were treated with metformin (Sigma-Aldrich, USA) in the drinking water (500 mg/kg/day) for 10 days. On day 7 the mice underwent 3 days UUO and were sacrificed on day 10. The kidneys were then processed for immunoblotting and quantitative polymerase chain reaction (qPCR) (n = 6). Due to surgical complications, one mouse was excluded from the UUO vehicle treated group. Animals were prepared in parallel for immunohistochemistry (n = 3–4). Sham mice, which were matched according to weight and age, had their ureters dissected free, but the ureters were not occluded. In order to find the optimal dose of metformin, we performed a pilot experiment in UUO mice with 4 different doses of metformin (50, 100, 300 and 500 mg/kg/day). The kidneys were processed for qPCR analysis (n = 4–5 mice in each group) to evaluate the effect of different doses on the expression of inflammatory, injury and oxidative stress markers. These data are included in Supplementary Figure 2 and demonstrate that metformin administered at a dose of 500 mg/kg/day significantly reduced renal inflammation, renal injury and oxidative stress in mice subjected to 3dUUO, whereas the lower doses generally had no significant effects.

#### Protocol 2

Male FVB or OCT1/2^−/−^ mice were treated as in protocol 1. The kidneys were processed for immunoblotting and qPCR (n = 6). Due to complications, one mouse was excluded from the WT vehicle treated group.

#### Protocol 3

Male C57BL/6 or AMPK-β^−/−^ (3 male, 1 female) mice were treated with metformin in the drinking water (500 mg/kg/day) for 10 days. On day 7 the mice underwent 3 days UUO and were then sacrificed on day 10 before the kidneys were processed for qPCR (n = 2–4).

At the day of sacrifice, blood was collected directly from the left ventricle of the heart. Blood was centrifuged and plasma levels of creatinine and urea were measured using a Roche Cobas 6000 analyzer (Roche Diagnostics, Switzerland). The plasma concentration of metformin was determined by analytical HPLC using a Dionex Ultimate 3000 system. Animal procedures in protocol 1 and 2 were performed in accordance with guidelines of the animal welfare policy at Aarhus University, Denmark and approved by the Animal Experiments Inspectorate, under the Danish Veterinary and Food Administration. Animal procedures in protocol 3 were performed in accordance with experimental protocols approved by the Institutional Animal Care and Use Committee at the University of Pittsburgh according to the NIH Guidelines for the Care and Use of Laboratory Animals.

### RNA isolation and qPCR analysis

Total RNA was isolated from mouse cortex with a NucleoSpin RNA II mini kit following the manufacturer’s instructions (Macherey-Nagel, Germany). Synthesis of cDNA was performed on 0.5 μg RNA using the RevertAid First Strand cDNA Synthesis KIT (Thermo Scientific, USA). For qPCR, 100 ng cDNA served as a template for PCR amplification using Brilliant SYBR Green QPCR Master Mix following the manufacturer’s instructions (Thermo Scientific).

### Electrophoresis and immunoblotting analysis for protocol 1 and 2

Renal cortex was homogenized in dissecting buffer (0.3 M sucrose, 25 mM imidazole, 1 mM EDTA, pH 7.2) containing the protease inhibitors: 8.5 μM leupeptin (Sigma-Aldrich) and 0.4 mM Pefabloc (Roche). The tissue was lysed in a tissuelyser LT (Qiagen, Germany) and centrifuged at 4,500 × *g* for 15 min. Gel samples were prepared from the supernatant by adding 2% SDS. The protein concentration of the homogenate was measured using a Pierce BCA protein assay kit (Roche) following the manufacturers manual.

Proteins were separated on 12% polyacrylamide gels (Protean II; Bio-Rad, Hercules, CA). Proteins were transferred to a nitrocellulose membrane (Amersham Pharmacia Biotech, Piscataway, NJ). Membranes were blocked with 5% milk in PBS-T (80 mM Na_2_HPO_4_, 20 mM NaH_2_PO_4_, 100 mM NaCl, 0.1 Tween 20, adjusted to pH 7.4). Following PBS-T wash, the membranes were incubated with primary antibodies overnight at 4 °C. Secondary horseradish peroxidase conjugated antibodies were incubated for 1 h and antigen-antibody complex was visualized using enhanced chemiluminescence system (Amersham Pharmacia Biotech, UK).

Primary antibodies: KIM-1 (R&D Systems, USA), HO-1 (ENZO, USA). Secondary antibodies: Polyclonal Goat Anti-Rabbit Immunoglobulin/HRP (DAKO, Denmark), Polyclonal Rabbit Anti Goat Immunoglobulin/HRP (DAKO). Target protein expression was normalized to total protein using stain-free technology developed by BIO-RAD Laboratories^55^. Total protein images are available under Supplementary Figure 3.

### Electrophoresis and immunoblotting analysis for protocol 3

Lysates were prepared using a Dounce homogenizer in lysis buffer containing 20 mM Tris.HCl pH 7.4, 50 mM NaCl, 50 mM NaF, 5 mM Na pyrophosphate, 250 mM sucrose, 1% Triton X-100, 1 mM DTT, 1 mM PMSF, and complete protease inhibitor cocktail (Roche). Homogenates were centrifuged at 16,000 × *g* for 30 min at 4 °C, and the protein concentration in the supernatants was measured using the Bradford method (Bio-Rad, Germany). Samples were separated by SDS-PAGE on a 4–12% gradient gel (Nu-PAGE; Invitrogen, USA) and electrically transferred to a PVDF membrane (Bio-Rad). The membrane was blocked in LI-COR Odyssey Blocking Buffer (TBS) for 1 h and then incubated in primary antibody. After a wash in TBS-0.1% Tween 20, the membrane was incubated for 1 h with secondary antibody in blocking buffer with 0.2% Tween 20. After a wash in TBS-0.1% Tween 20, immunoreactive proteins were detected by an Odyssey^®^ Fc Imaging System (LI-COR). If the membrane was to be probed with another primary antibody, antibody bound to the membrane was stripped by an incubation in NewBlot™ IR stripping solution (LI-COR) for 15 min. Quantification of Western blots was performed by densitometry with analysis using Image Studio Lite Ver 5.2 software (LI-COR, USA).

Primary antibodies: AMPK-α, AMPK-α phospho-Thr172 (Cell Signaling Technology, USA), AMPK β1/2 (Epitomics, USA) and β-actin (Sigma-Aldrich). Secondary antibodies: IRDye^®^680RD Goat anti-Rabbit or IRDye^®^ 800CW Goat anti-Mouse secondary antibody (LI-COR). Target protein expression was normalized to beta-actin expression.

### Histology

Mice were fixed by retrograde perfusion via the left ventricle with 4% paraformaldehyde in 0.01 M PBS buffer. Next, kidneys were post-fixed for an additional hour and washed in 0.01 M PBS buffer. Fixed kidneys were dehydrated, embedded in paraffin, and cut into 2-μm sections on a rotary microtome (Leica Microsystems, Denmark). Sections were stained with hematoxylin and eosin to analyze the degree of tubular damage. The tubular luminal area of each section was measured using the image analysis software ImageJ (National Institutes of Health, USA). Representative pictures of the renal cortex are shown in 40x magnification.

### Immunofluorescence labeling

For immunolabeling, the sections were deparaffinized overnight in xylene, rehydrated in 99% to 70% ethanol, and boiled in TEG-buffer to improve target availability. Non-specific binding to free aldehyde groups was blocked with 50 mM NH_4_Cl in PBS followed by incubation in PBS containing 1% BSA, 0.2% gelatin, and 0.05% saponin. The tissue slides were incubated with primary antibody KIM-1 (R&D Systems) diluted in PBS containing 0.1% BSA and 0.3% Triton X-100, in a humidified chamber overnight at 4 °C. After washing in 0.1% BSA, 0.2% gelatin, 0.05% saponin in PBS, the tissue slides were incubated with Alexa fluor 568 secondary antibody and DAPI (Sigma) for nuclear staining. Tissue slides were mounted using Slowfade Light Antifade (Invitrogen, USA). Images were taken to overlap and merged in Photoshop© CS5.

### TUNEL assay

TUNEL assay was performed using the ApopTag Plus Fluorescein *In Situ* Apoptosis Detection Kit following the manufacturer’s protocol (Millipore, USA).

### Radiochemistry

[^11^C]-metformin was prepared as described earlier^56^ Briefly, [^11^C]-Methyl iodide was reacted with 1-methylbiguanide dissolved in acetone and the crude product was purified by reverse phase HPLC. The final product (0.2–1.0 GBq) contained 0.1–0.5 microgram/ml metformin in aqueous (NH_4_)_2_HPO_4_ (100 mM, pH 7).

### MicroPET

Female FVB WT and FVB OCT1/2^−/−^ mice (age: 13–15 weeks). WT (n = 6) and OCT1/2^−/−^ mice (n = 4) underwent PET- and anatomical MRI-scans using Mediso nanoScan PET/MR (Mediso Ltd., Hungary). These mice have earlier been used in another study which has been publiched^57^. Anesthesia was maintained with isoflurane. A catheter was placed into the tail vein and a single bolus of [^11^C]-metformin (5.2 ± 2.5 MBq/body) was injected via the catheter, followed by 60 min dynamic PET- and 30 min MR-imaging. Body temperature was kept at 36–37 °C. These studies were performed in accordance with guidelines of the animal welfare policy at Aarhus University, Denmark and approved by the Animal Experiments Inspectorate, under the Danish Veterinary and Food Administration.

### PET-image analysis

Dynamic PET data were reconstructed as a 3DOSEM algorithm (Tera-Tomo 3D, full detector model and normal regularization; Mediso Ltd., Hungary) with 4 iterations and 6 subsets, voxel size 0.4 × 0.4 × 0.4 mm^3^. Data were corrected for dead-time and decay, and for randoms’ using a delayed coincidence window. No corrections were made for attenuation and scatter. The dynamic PET scans were reconstructed as 30 frames increasing in duration from 5 sec to 10 min. Multiple regions of interest were placed on coronal slices in the organ of interest using PMOD version 3.5 (PMOD Technologies Ltd, Switzerland) creating a volume of interest (VOI). An image-derived input function was generated by averaging images from the first 20 sec and placing a circle with a diameter of 15 pixels on the 6 most intensive slices in the heart (68 μL). VOI in renal parenchyma were drawn in both kidneys on PET images averaging all frames. MR-images were used for defining size and demarcation of the VOIs. Positioning of VOIs was controlled in each time frame. Time-activity curves were generated from the VOIs and expressed as an average of the two kidneys. Kidney-to-blood ratio was calculated by dividing the renal parenchymal concentration of [^11^C]-metformin by the blood concentration at each time point for each animal. Area under the kidney-to-blood ratio curve was used to compare exposure of [^11^C]-metformin in renal parenchyma between the two groups. Due to pathological tracer accumulation in the renal parenchyma, one WT animal was excluded from further analysis.

### Statistical analysis

Multiple comparisons between groups were performed using two-way ANOVA followed by Tukey’s post hoc tests. To elucidate differences among metformin treatments, a Kruskal-Wallis test followed by a Dunn’s multiple comparison test were used. In the PET study the results were analyzed by a Student’s t-test. Descriptive statistics are presented as mean values ± SEM. P < 0.05 was considered statistically significant indicated by *P < 0.01 by **P < 0.001 by *** and not significant by ns.

## Additional Information

**How to cite this article**: Christensen, M. *et al*. Renoprotective Effects of Metformin are Independent of Organic Cation Transporters 1 & 2 and AMP-activated Protein Kinase in the Kidney. *Sci. Rep.* **6**, 35952; doi: 10.1038/srep35952 (2016).

**Publisher’s note:** Springer Nature remains neutral with regard to jurisdictional claims in published maps and institutional affiliations.

## Supplementary Material

Supplementary Information
