# Supplementary material for: Renoprotective Effects of Metformin are Independent of Organic Cation Transporters 1 & 2 and AMP-activated Protein Kinase in the Kidney
Source: Sci Rep. 2016 Oct 26;6:35952. doi: 10.1038/srep35952 (PMC5080611; doi:10.1038/srep35952)
Supplement: Supplementary Information [file srep35952-s1.pdf]

**Supplementary data from the original manuscript by Christensen et al entitled:**

# Renoprotective Effects of Metformin are Independent of Organic Cation Transporters 1 & 2 and AMP-activated Protein Kinase in the Kidney.

Michael Christensen[1], Jonas B. Jensen[1,2], Steen Jakobsen[2], Niels Jessen[1], Jørgen Frøkiær[1,2], Bruce E. Kemp[3], Allison L. Marciszyn[4], Hui Li[4,5], Núria M. Pastor-Soler[4,5], Kenneth R. Hallows[4,5] and Rikke Nørregaard[1]*.

| | SHAM | SHAMmet | UUO | UUOmet |
|---|---|---|---|---|
| **Metformin (ng/mL)** | nd | 2200 ± 36 | nd | 3289 ± 47 |
| **Creatinine (µmol/L)** | 8.17 ± 0.28 | 8.17 ± 0.37 | 11.83 ± 0.68* | 11.67 ± 0.77* |
| **Urea (mmol/L)** | 8.52 ± 0.55 | 7.15 ± 0.34 | 10.83 ± 0.49* | 9.67 ± 0.43* |

**Supplementary table 1: Effect of metformin on renal function in response to 3 days unilateral ureteral obstruction.** Plasma metformin, creatinine and urea levels in sham and UUO mice treated with and without metformin.

| | WT | WTmet | OCT1/2KO | OCT1/2KOmet |
|---|---|---|---|---|
| **Metformin (ng/mL)** | nd | 2892 ± 335 | nd | 4614 ± 783 |
| **Creatinine (µmol/L)** | 8.6 ± 0.67 | 8.5 ± 0.39 | 9.5 ± 0.31* | 12.4 ± 0.61*# |
| **Urea (mmol/L)** | 12.08 ± 0.67 ns | 12.27 ± 0.27 | 9.00 ± 0.53* | 9.16 ± 0.63* |

**Supplementary table 2: Effect of metformin on renal function in response to 3 days unilateral ureteral obstruction in OCT1/2 KO mice.** Plasma metformin, creatinine and urea levels in sham and UUO OCT1/2 KO mice treated with and without metformin.

**Supplementary table 3: QPCR primers sequences.**

| Target | Forward primer | Reverse primer |
|---|---|---|
| **TNFα** | 5’- AGGCTGCCCCGACTACGT-3’ | 5’-GACTTTCTCCTGGTATGAGATAGCAAA-3’ |
| **IL-6** | 5´- GATGCTACCAAACTGGATATAATC-3’ | 5’-GGTCCTTAGCCACTCCTTCTGTG-3’ |
| **IL-1β** | 5’-CAGGCAGGCAGTATCACTCA-3’ | 5’-TGTCCTCATCCTGGAAGGTC-3’ |
| **F4/80** | 5’-TGCTAGTGGAGGCAGTGATG-3’ | 5’-CTGTATTCAACCAGCAGCGA-3’ |
| **Itgax** | 5’-ACTGACCTGGTCCTGATTGG-3’ | 5’-CAGCACCTCTGTTCTCCTCC-3’ |
| **Mac2** | 5’-GCTTATCCTGGCTCAACTG C-3’ | 5’-TTCACTGTGGCCCATGATTGT-3’ |
| **KIM1** | 5’-CGGTACAACTTAAAGGGGCA-3’ | 5’-GACGTGTGGGAATCTCTGGT-3’ |
| **Nqo1** | 5’-CAGATCCTGGAAGGATGGAA-3’ | 5’-TCTGGTTGTCAGCTGGAATG-3’ |
| **Vcam1** | 5’-GTGGTGCTGTGACAATGACC-3’ | 5’-ACGTCAGAACAACCGAATCC-3’ |
| **Icam1** | 5’-TCCAATTCACACTGAATGCC-3’ | 5’-GTCTGCTGAGACCCCTCTTG-3’ |
| **MCP1** | 5’-CAAGAAGGAATGGGTCCAGA-3’ | 5’-GTGCTGAAGACCTTAGGGCA-3’ |
| **OCT3** | 5’-AATATCCTGTTTCGGCGTTG-3’ | 5’-TCACGATCACGAAGCAAGTC-3’ |
| **Pmat** | 5’-GTCTTCTCGCTGCTAATGGG-3’ | 5’-ATGGTGTTTCCTGCCAGTTC-3’ |
| **Mate1** | 5’-CTGCTCTTCAGACAGGACCC-3’ | 5’-TGACAAGGTTAGCTGCGATG-3’ |
| **Mate2** | 5’-TCTTCCTGCAGCGTTCCTAT-3’ | 5’-CTCCACTCCTGGAAGCACTC-3’ |
| **C-Myc** | 5’-CAACGTCTTGGAACGTCAGA-3’ | 5’-TCGTCTGCTTGAATGGACAG-3’ |

# Supplementary figure 1

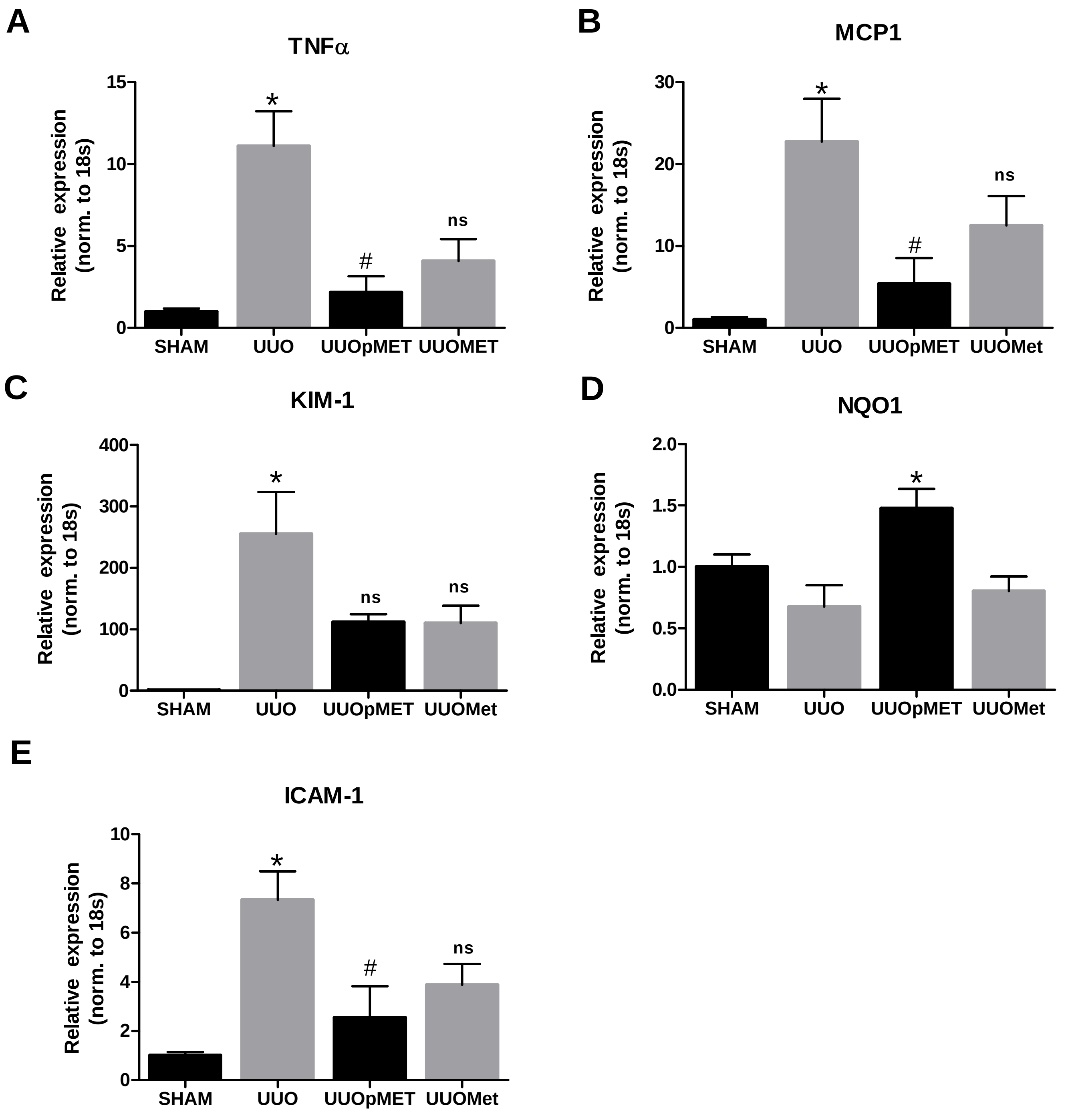

**Supplementary figure 1: The effect of metformin in mice subjected to 3 days unlateral ureteral obstruction (UUO).**

UUO mice were treated with metformin (500 mg/kg/day) after UUO damage. The kidneys were processed for qPCR analysis (n = 4-5 mice in each group) to evaluate the different doses on the expression of inflammatory, injury and oxidative stress markers. Regulation of the inflammatory and injury markers (A) TNFα, (B) MCP1, (C) KIM1, (D) NQO1 and (F) ICAM1 in mice subjceted to 3dUUO and metformin treatment normalized to ribosomal 18S RNA. Each bar represents the mean ± SEM. *P<0.05 compared to the sham group. # P<0.05 compared to the UUO group. Data demonstrated that metformin administrated at a dose of 500 mg/kg/day after induction of UUO had some renoprotective effects in mice subjected to 3dUUO.

UUOpMET: UUO mice were pretreated with metformin. UUOMet: UUO mice were treated with metformin after UUO damage.

# Supplementary figure 2

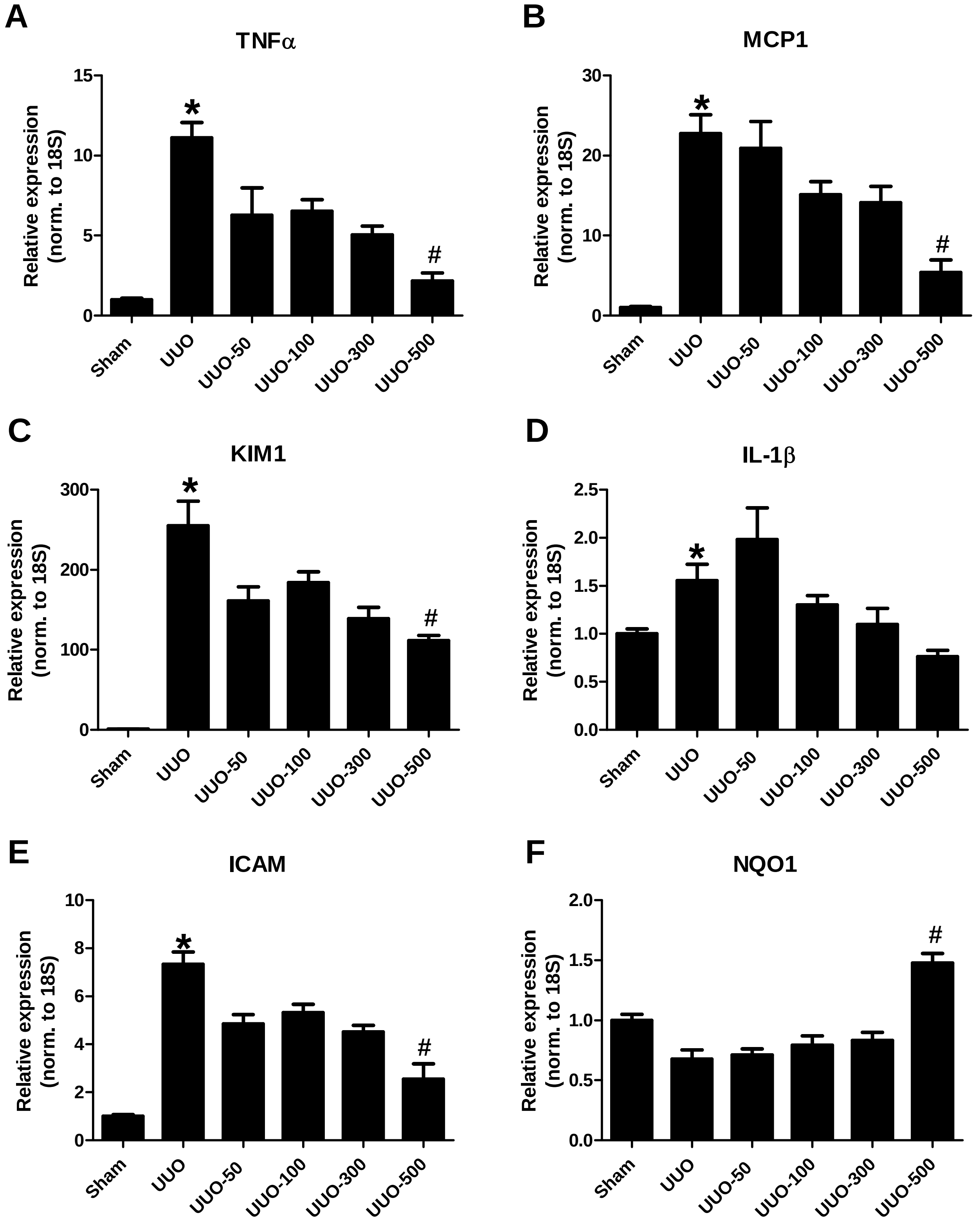

**Supplementary figure 2: Dose-response experiment in mice subjected to 3 days unilateral ureteral obstruction (UUO) with different doses of metfomin.**

UUO mice were administrated 4 different doses of metformin (50, 100, 300 and 500 mg/kg/day). The kidneys were processed for qPCR analysis (n = 4-5 mice in each group) to evaluate the different doses on the expression of inflammatory, injury and oxidative stress markers. Regulation of the inflammatory and injury markers (A) TNFα, (B) MCP1, (C) KIM1, (D) IL-1β, (E) ICAM and the oxidative stress marker (F) NQO1 in mice subjected to 3dUUO and metformin treatment normalized to ribosomal 18S RNA. Each bar represents the mean ± SEM. *P<0.05 compared to the sham group. # P<0.05 compared to the UUO group.

Data demonstrated that metformin administrated at a dose of 500 mg/kg/day reduced significantly renal inflammation, renal injury and oxidative stress in mice subjected to 3dUUO whereas the lower doses had no significant effect.

# Supplementary figure 3

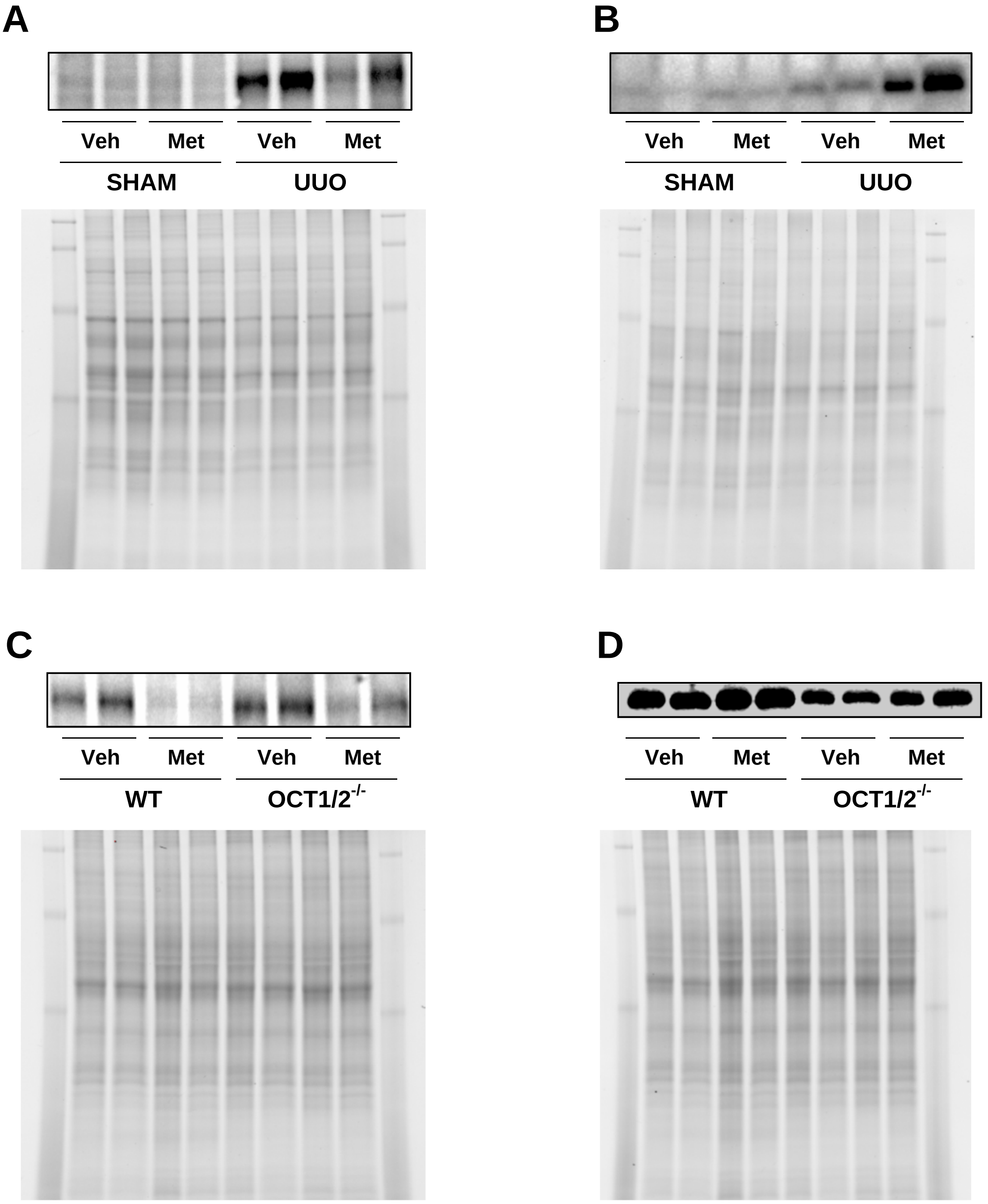


**Supplementary figure 3 – Total protein gels for western blots in figure 3, 4 and 5.** Western blots with corresponding total proteins. A: KIM-1 from figure 3, B: HO-1 from figure 4, C: KIM-1 from figure 5 and D: HO-1 from figure 5.
